# A global dataset of terrestrial evapotranspiration and soil moisture dynamics from 1982 to 2020

**DOI:** 10.1038/s41597-024-03271-7

**Published:** 2024-05-03

**Authors:** Kun Zhang, Huiling Chen, Ning Ma, Shasha Shang, Yunquan Wang, Qinglin Xu, Gaofeng Zhu

**Affiliations:** 1 School of Geospatial Engineering and Science, Sun Yat-Sen University, Zhuhai, China; 2 School of Biological Sciences, The University of Hong Kong, Hong Kong, China; 3 College of Geography and Environmental Science, Zhejiang Normal University, Jinhua, China; 4 Key Laboratory of Water Cycle and Related Land Surface Processes, Institute of Geographic Sciences and Natural Resources Research, Chinese Academy of Sciences, Beijing, China; 5 Tianjin Key Laboratory of Water Resources and Environment, Tianjin Normal University, Tianjin, China; 6 School of Environmental Studies, China University of Geosciences, Wuhan, China; 7 The 404 Company Limited, CNNC, Lanzhou, China; 8 College of Earth and Environmental Sciences, Lanzhou University, Lanzhou, China

**Keywords:** Hydrology, Hydrology

## Abstract

Quantifying terrestrial evapotranspiration (ET) and soil moisture dynamics accurately is crucial for understanding the global water cycle and surface energy balance. We present a novel, long-term dataset of global ET and soil moisture derived from the newly developed Simple Terrestrial Hydrosphere model, version 2 (SiTHv2). This ecohydrological model, driven by multi-source satellite observations and hydrometeorological variables from reanalysis data, provides daily global ET-related estimates (e.g., total ET, plant transpiration, soil evaporation, intercepted evaporation) and three-layer soil moisture dynamics at a 0.1° spatial resolution. Validation with *in-situ* measurements and comparisons with mainstream global ET and soil moisture products demonstrate robust performance of SiTHv2 in both magnitude and temporal dynamics of ET and soil moisture at multiple scales. The comprehensive water path characterization in the SiTHv2 model makes this seamless dataset particularly valuable for studies requiring synchronized water budget and vegetation response to water constraints. With its long-term coverage and high spatiotemporal resolution, the SiTHv2-derived ET and soil moisture product will be suitable to support analyses related to the hydrologic cycle, drought assessment, and ecosystem health.

## Background & Summary

Water and energy exchanges between the land and atmosphere play a critical role in shaping the climate^1,2^. Evapotranspiration (ET) serves as a crucial nexus in the intricate global water and carbon cycles^3–5^, not only maintaining water balance but also affecting surface energy balance^6–8^. As the primary mechanism for water transfer from soil to atmosphere, ET consumes over 50% of available radiation and returns 60% of annual terrestrial precipitation^9,10^. Accurate estimation of ET and its different components (i.e., plant transpiration, soil evaporation, and canopy interception evaporation) is thus essential for understanding global energy balance and water cycle, particularly when separating vegetation feedbacks in the context of a changing climate.

Terrestrial ET is theoretically governed by two terms: atmospheric demand, which determines the potential ability to accept water vapor^11^, and local water supply, which regulates the actual amount of ET^12^. Among various water transfer mechanisms on Earth’s surface, soil moisture plays a crucial role in impacting actual ET by directly representing soil water availability^13,14^. Consequently, accurate estimates of both ET and soil moisture mutually complement each other. Although process-based ET models have proven effective in quantifying terrestrial ET^15^, the majority of these models do not comprehensively integrate underlying ecohydrological processes, such as canopy interception^16^, groundwater table dynamics^17,18^, and root water uptake^19,20^.

The Simple Terrestrial Hydrosphere (SiTH) model^21^ integrates the eco-hydrological process with remotely sensed information to characterize the water pathway within the groundwater-soil-plant-atmosphere continuum (GSPAC)^22^, which demonstrated favorable performance in modeling ET across various ecosystems^23–25^. Recent evidence has shown that groundwater table dynamics significantly affect root zone soil moisture^26,27^, surface runoff production^28,29^, land-atmosphere interactions^30,31^, and regional climate^32,33^. However, most research focused on regional scales, while simple and effective expressions of groundwater effects on vegetation at the global scale have been lacking. To address this, we considered potential deeper layer recharge in soil moisture based on the saturated hydraulic conductivity for different soil properties^34^. It should be noted that root distribution is important in influencing vegetation transpiration^35^, while existing models have predominantly focused on the vertical distribution of roots with relatively shallow burial depths. Meanwhile, the comprehensive effect of vertical root distribution and multi-layer soil water content on plant transpiration are not well represented in current models^21^. As a result, previous models with shallow root depths tend to underestimate vegetation drought resistance and overestimate the sensitivity of plant transpiration to water stress. Thus, the comprehensive effect of vertical root distribution and multi-layer soil water content are synchronously considered in the updated SiTH model (SiTHv2)^20^. Moreover, we modified the critical parameter of soil moisture in SiTHv2 to better represent plant water stress by considering different vegetation heights and varied environmental conditions. Notably, the SiTHv2 model uses satellite-observed vegetation status, including vegetation greenness^36^ and optical depth^37^, to characterize plant growth and health.

**Fig. 1:**
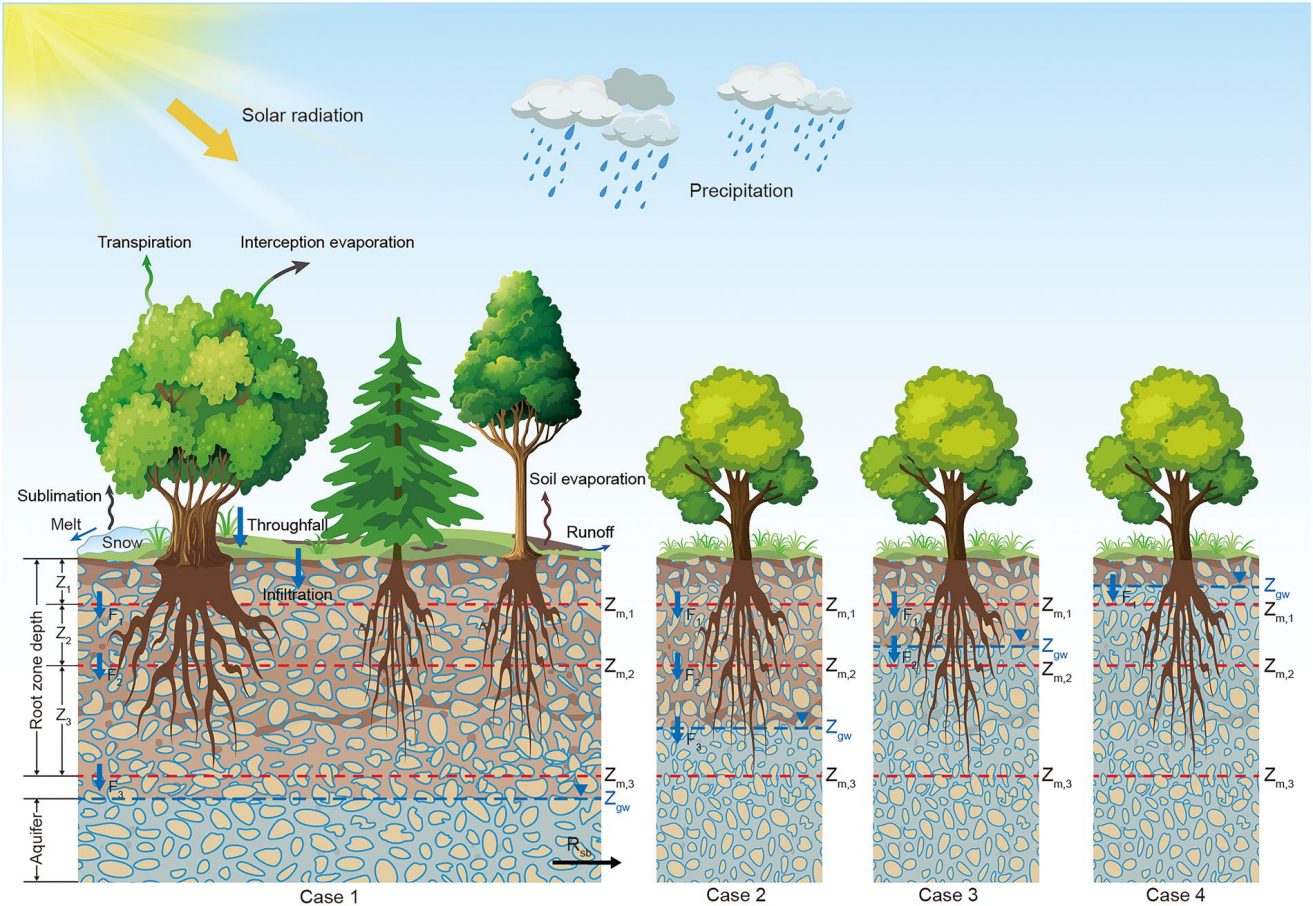
The conceptual diagram of the hydrological process in the SiTHv2 model. Different cases indicate the groundwater table (*z*_gw_) dynamically related with potential root zone depth.

In this study, we present a novel dataset of global terrestrial ET (including different components) and soil moisture generated using the SiTHv2 model. This dataset provides daily estimates at a spatial resolution of 0.1°, with seamless global land coverage. The conceptual graph of the SiTHv2 model is illustrated in Fig. 1, and the model description and data production procedure are presented schematically in the Methods section. The availability of this dataset will provide essential support and valuable insights for scientific research in the domains of land-atmosphere interactions, ecohydrological modeling, and global change studies.

## Methods

### Forcing data and preprocessing

#### Remote sensing data

Vegetation dynamics play a crucial role in the SiTHv2 model, particularly in the partitioning scheme for available energy. We utilized the leaf area index (LAI) from the European Geoland2/BioPar project Version 2 (GEOV2)^38^ based on the retrievals of the Advanced Very High-Resolution Radiometer (AVHRR). This product offers global LAI observations every 10 days at a 0.05° spatial resolution and has been proven as a reliable satellite-based product for providing long-term vegetation variables with global coverage. Additionally, we employed vegetation optical depth (VOD) to characterize the actual vegetation water content from the VOD Climate Archive (VODCA)^37^. This product based on microwave observations in various spectral bands, provides daily VOD estimates at a 0.25° spatial resolution. We selected the Ku-band due to its extensive data availability. Furthermore, we used the Global Land Surface Satellite (GLASS) product^39^ to obtain surface broadband albedo and emissivity observations with 8-day intervals and a spatial resolution of 0.05°. Specifically, the GLASS02B03 dataset informed the black-sky and white-sky albedo, while the GLASS03B02 dataset contributed to broadband emissivity.

#### Hydrometeorological data

Hydrometeorological variables serve as essential inputs for the SiTHv2 model. We acquired daily air temperature and air pressure data from the Multi-Source Weather (MSWX) product^40^, a bias-corrected meteorological dataset with global coverage and a spatial resolution of 0.1° since 1979. In terms of radiation, we used downward shortwave radiation and downward longwave radiation from the land component of the fifth-generation European reanalysis data (ERA5-Land, hereafter referred as ERA5L)^41^. We also utilized skin temperature from ERA5L, combined with satellite-based broadband emissivity, to calculate upward longwave radiation. Moreover, to calculate the actual albedo (i.e., blue-sky albedo), we assumed the blue-sky albedo is linearly weighted between black-sky and white-sky albedo. The weight coefficient is the sky scattering ratio, derived from the mean surface direct shortwave radiation and mean surface downward shortwave radiation from ERA5^42^. Consequently, global long-term seamless surface net radiation can be calculated using the balance equation of the four radiation components. Additionally, the precipitation data used in this study was obtained from the ERA5L product. It is noteworthy that the original temporal interval of ERA5 and ERA5L are hourly, yet their spatial resolutions differ, with ERA5 at 0.25° and ERA5L at 0.1°.

#### Auxiliary data

Land cover/use dynamics also significantly influence the estimation of terrestrial ET, considering different biological effects (e.g., plant height and root depth) in the parameterization scheme. Current satellite-based land cover products are hard to provide temporal dynamic information over a long-term period. Thus, we obtained annual land cover dynamics from the Historic Land Dynamics Assessment+ (HILDA+) product^43^, a long-term land cover/use product combining multiple open data streams, including remote sensing, reconstructions, and statistics. Moreover, the global soil type used in the SiTHv2 model was acquired from the Harmonized World Soil Database (HWSD) v1.2, combining available soil information from regional and national institutes worldwide and offering a soil raster database with a 30-arc-second resolution^44^.

To reconcile the varying spatial resolutions and temporal intervals of the aforementioned multiple data sources in the SiTHv2 model, we uniformly resampled the spatial resolution to 0.1° using the bilinear approach and filled the temporal gap to the daily scale based on spline interpolation.

### Validation and comparison data

We evaluated the accuracy of SiTHv2 estimates using both *in-situ* observations and grid-based ET and soil moisture products. Specifically, we validated the total ET estimates of SiTHv2 using latent heat flux measurements from 175 global eddy covariance (EC) stations with a daily interval^45,46^. These EC stations encompass nine major plant functional types (PFTs) under various climate conditions, including croplands (CRO), deciduous broadleaf forests (DBF), evergreen broadleaf forests (EBF), evergreen needleleaf forests (ENF), grasslands (GRA), mixed forests (MF), open shrublands (OSH), savannas (SAV), and wetlands (WET). Moreover, we employed pre-processing method according to previous study^10^ and ensure the surface energy closure at least above 70%^47^ (Figure S1). Five ET products were selected for comparison at both basin and global scales, including ET estimates from: (1) the Global Land Evaporation Amsterdam Model (GLEAM)^48^; (2) the calibration-free complementary relationship (CR) model^49^; (3) the FluxCOM product^50^; (4) the Global Land Data Assimilation System (GLDAS-Noah)^51^; and (5) ERA5L^41^. Furthermore, we utilized ET simulations from 20 Earth System Models (ESMs) in the Coupled Model Intercomparison Project Phase 6 (CMIP6)^52^ to compare SiTHv2 ET estimates concerning annual variation and magnitude of total ET volume at a global level.

We generated water-balanced ET (*ET*_wb_) from the residual of the basin water balance equation (precipitation minus runoff and changes in total water storage) to validate SiTHv2-based ET estimates at a basin scale. A total of 49 basins were selected that covering a broad range of climate zones globally. The precipitation data used to calculate *ET*_wb_ obtained from the gauge-based Global Precipitation Climatology Center (GPCC) Full Data Monthly Product^53^. However, for the Continental United States (CONUS), we used the Parameter-Elevation Regressions on Independent Slopes Model (PRISM) precipitation data^54^. Runoff observations from hydrological stations for the majority of basins, excluding those in the CONUS and China, were obtained from the Global Runoff Data Center (GRDC). Runoff data for basins within the CONUS and China were sourced from the United States Geological Survey and the China Sediment Bulletin, respectively. Basin-scale changes in total water storage were derived from Gravity Recovery and Climate Experiment (GRACE) terrestrial water storage anomaly products^55^. To minimize uncertainties arising from noise terms in different solutions, we calculated the average of three GRACE products processed by the Geoforschungs Zentrum Potsdam, the Center for Space Research at the University of Texas, Austin, and the NASA Jet Propulsion Laboratory. For more details on the *ET*_wb_ calculation, please refer to the authors’ previous studies^56,57^.

To validate the soil moisture estimated by the SiTHv2 model, we selected 12 sites from FLUXNET offering daily *in-situ* measurements of soil water content (SWC). We also compared SiTHv2-derived soil moisture with a global soil moisture dataset, SoMo.ml^58^, which is based on the Long Short Term Memory (LSTM) network and trained with abundant *in-situ* measurements from over 1,000 stations. SoMo.ml has been considered to perform well in both spatial distribution and temporal dynamics of soil moisture, and can serve as a benchmark to evaluate modelled and remotely sensed data^58^. Furthermore, we utilized four types of microwave-based satellite products for soil moisture comparison, comprising: L-band (1.4 GHz) products obtained from the Soil Moisture Ocean Salinity (SMOS) at the Barcelona Expert Centre (BEC)^59^ and the Soil Moisture Active Passive (SMAP) Level-3 products^60^; as well as C-band (6.9 and 7.3 GHz) products from the Advanced SCATterometer (ASCAT)^61^ and the Advanced Microwave Scanning Radiometer 2 (AMSR2) of Japan Aerospace Exploration Agency (JAXA)^62^. These products offer a spatial resolution of 0.25°, with the exception of the SMAP L3, which features an enhanced spatial resolution of 9 km.

The selected validation and comparison product offers independent reference for evaluating the ET and soil moisture estimates derived from the SiTHv2 model. A summary of all mentioned products can be found in Table 1.

**Table 1:** Summary of the validation/comparison datasets used in this study.

Product	Variable	Version/Type	Spatial resolution	Interval(3)	Time span
FLUXNET2015	ET & SWC	N/A	*In-situ*	Daily	Vaied with sites
GLEAM	ET	v3.7a	0.25°	Monthly	1980-
CR	ET	v1.0	0.25°	Monthly	1982–2016
FluxCOM	ET	RS_METEO(1)	0.5°	Monthly	2001–2013
GLDAS-Noah	ET	v2.0	0.25°	Monthly	1982–2014
ERA5-Land	ET	N/A	0.1°	Monthly	1950-
CMIP6	ET	20 ESMs(2)	0.5°	Monthly	1979–2014
SoMo.ml	SWC	N/A	0.25°	Daily	2001–2019
SMAP	SWC	Enhanced L3	9 km	Daily	2015-
SMOS	SWC	BEC L3	0.25°	Daily	2011-
ASCAT	SWC	v7	0.25°	Daily	2007-
AMSR2	SWC	JAXA	0.25°	Daily	2012-

Note: (1) All the ensembled latent heat flux estimates of 36 members with energy balance correction; (2) Details of the 20 ESMs (historical period) are provided in Table S1 in Supplementary; (3) The time intervals listed in the table represent only the types used in this study, while some products may have a higher temporal resolution.

### SiTHv2 model description

The SiTHv2 model is an updated version of the SiTH model, which is developed for modeling the water pathway within the GSPAC (Fig. 1). Evapotranspiration (ET) and soil moisture (also known as soil water content, SWC) are two crucial outputs generated by the model. Specifically, the total ET in the SiTHv2 is the sum of soil evaporation (*E*_s_), plant transpiration (*T*_r_), and canopy interception evaporation (*E*_i_). Each item is derived from different kinds of constraints from potential transpiration and evaporation for the canopy and soil surface, respectively.

1 $$ET={E}_{{\rm{i}}}+{E}_{{\rm{s}}}+{T}_{{\rm{r}}}$$

2 $${E}_{{\rm{i}}}={f}_{{\rm{wet}}}\cdot {E}_{{\rm{pc}}}$$

3 $${E}_{{\rm{s}}}={f}_{{\rm{sm}}}\cdot {E}_{{\rm{ps}}}$$

4 $${T}_{{\rm{r}}}={f}_{{\rm{v}}}\cdot {f}_{{\rm{t}}}\cdot \left[\mathop{\sum }\limits_{i=1}^{n}\left({f}_{{\rm{smv}},{\rm{i}}}\cdot {T}_{{\rm{ps}},{\rm{i}}}\right)+\mathop{\sum }\limits_{i=1}^{n}\left({T}_{{\rm{pg}},{\rm{i}}}\right)\right]$$

where *n* is the total number of soil layers, which is set as three in SiTHv2; the *f*-functions are the different constraints on the potential evapotranspiration for canopy (*E*_pc_) and soil surface (*E*_ps_), where the *f*_wet_ is the relative wetness of canopy surface; the *f*_sm_ is the soil moisture constraint of bare soil evaporation; the *f*_smv, i_ is the soil moisture constraint of plant transpiration in the unsaturated zone at *i*^th^ soil layer; the *f*_v_ and *f*_t_ are the vegetation water content and temperature constraint on plant health, respectively. Notably, the characterization of potential transpiration rate (*T*_p_) is comprised of both unsaturated and saturated layers when the groundwater table extends to the *i*^th^ soil layer, denoted as *T*_ps, i_ and *T*_pg, i_, respectively (see different cases in Fig. 1). The calculation of *E*_pc_ and *E*_ps_ in the SiTHv2 model are based on the Priestly-Taylor equation^63^, while the *T*_p_ can be derived by the residual of *E*_pc_ after intercepted consumption.

5 $${E}_{{\rm{ps}}}=\alpha \cdot \frac{\Delta }{\Delta +\gamma }\cdot \frac{\left({R}_{{\rm{ns}}}-G\right)}{\lambda }$$

6 $${T}_{{\rm{p}}}=\left(1-{f}_{{\rm{wet}}}\right)\cdot {E}_{{\rm{pc}}}=\left(1-{f}_{{\rm{wet}}}\right)\cdot \alpha \cdot \frac{\Delta }{\Delta +\gamma }\cdot \frac{{R}_{{\rm{nc}}}}{\lambda }$$

where *α* is the Priestly-Taylor coefficient and set to 1.26 in this study; Δ is the slope of the saturated vapor pressure curve (kPa °C^−1^); *γ* is the psychrometric constant and set to 0.066 (kPa °C^−1^); *λ* is the latent heat of evaporation (MJ kg^−1^); *R*_ns_ and *R*_nc_ are the net radiation (*R*_n_) allocated to the bare soil and canopy surface, respectively. *R*_ns_ is complementary with *R*_nc_, which can be derived by the Beer law:

7 $${R}_{{\rm{ns}}}={e}^{\left(-{k}_{{\rm{Rn}}}\cdot LAI\right)}\cdot {R}_{{\rm{n}}}$$

where *k*_Rn_ is the extinction coefficient and is set to 0.6 in this study; *LAI* is the leaf area index. Thus, the potential transpiration for different soil layers (*T*_p, i_) can be derived from the *T*_p_ by considering the vertical distributions of plant roots and the soil water content at different depths:

8 $${T}_{{\rm{p}},{\rm{i}}}=\frac{{r}_{{\rm{i}}}\cdot {\left({\bar{\theta }}_{{\rm{i}}}/{\theta }_{{\rm{s}}}\right)}^{b}}{{\sum }_{i=1}^{n}\left[{r}_{{\rm{i}}}\cdot {\left({\bar{\theta }}_{{\rm{i}}}/{\theta }_{{\rm{s}}}\right)}^{b}\right]}\cdot {T}_{{\rm{p}}}$$

where *θ*_s_ is the saturated soil water content; $\overline{{\theta }_{{\rm{i}}}}$ is the layer mean water content depend on the fraction of unsaturated zone for each soil layer; *b* is a soil parameter (dimensionless) depends on soil properties^21^; *r*_i_ presents the vertical root density, which is described by the linear dose response model^64^:

9 $${r}_{{\rm{i}}}=\frac{1}{1+{\left({z}_{{\rm{m}},{\rm{i}}}/{D}_{50}\right)}^{c}}-\frac{1}{1+{\left({z}_{{\rm{m}},{\rm{i}}-1}/{D}_{50}\right)}^{c}}$$

where the depth at which 50% of the root mass is located is referred to as *D*_50_; *z*_m, i_ is the bottom depth of the *i*^th^ layer; *c* is the shape parameter derived from the logarithmic relation between *D*_50_ and *D*_95_, which is the depth at which 95% of the root mass is located. Thus, the variables *T*_ps, i_ and *T*_pg, i_ can be further obtained from the *T*_p, i_ under different conditions of groundwater depths (*z*_gw_) and soil water content for different layers.

10 $${T}_{{\rm{ps}},{\rm{i}}}=\left\{\begin{array}{l}0\\ \frac{\left({z}_{{\rm{gw}}}-{z}_{{\rm{m}},{\rm{i}}-1}\right)\cdot {\theta }_{{\rm{i}}}}{\left({z}_{{\rm{gw}}}-{z}_{{\rm{m}},{\rm{i}}-1}\right)\cdot {\theta }_{{\rm{i}}}+\left({z}_{{\rm{m}},{\rm{i}}}-{z}_{{\rm{gw}}}\right)\cdot {\theta }_{{\rm{s}}}}\\ {T}_{{\rm{p}},{\rm{i}}}\end{array}\right..{T}_{{\rm{p}},{\rm{i}}}\begin{array}{l}{z}_{{\rm{gw}}}\le {z}_{{\rm{m}},{\rm{i}}-1}\\ {z}_{{\rm{m}},{\rm{i}}-1} < {z}_{{\rm{gw}}} < {z}_{{\rm{m}},{\rm{i}}}\\ {z}_{{\rm{gw}}}\ge {z}_{{\rm{m}},{\rm{i}}}\end{array}$$

11 $${T}_{{\rm{pg}},{\rm{i}}}=\left\{\begin{array}{l}{T}_{{\rm{p}},{\rm{i}}}\\ \frac{\left({z}_{{\rm{m}},{\rm{i}}}-{z}_{{\rm{gw}}}\right)\cdot {\theta }_{{\rm{s}}}}{\left({z}_{{\rm{gw}}}-{z}_{{\rm{m}},{\rm{i}}-1}\right)\cdot {\theta }_{{\rm{i}}}+\left({z}_{{\rm{m}},{\rm{i}}}-{z}_{{\rm{gw}}}\right)\cdot {\theta }_{{\rm{s}}}}\\ 0\end{array}\right..{T}_{{\rm{p}},{\rm{i}}}\begin{array}{l}{z}_{{\rm{gw}}}\le {z}_{{\rm{m}},{\rm{i}}-1}\\ {z}_{{\rm{m}},{\rm{i}}-1} < {z}_{{\rm{gw}}} < {z}_{{\rm{m}},{\rm{i}}}\\ {z}_{{\rm{gw}}}\ge {z}_{{\rm{m}},{\rm{i}}}\end{array}$$

In addition, the *f*-functions for different constraints in SiTHv2 can be calculated as follows:

12 $${f}_{{\rm{wet}}}=\min \left\{\chi \cdot \frac{{S}_{{\rm{c}}}}{{T}_{{\rm{p}}}},1\right\}$$

13 $${f}_{{\rm{sm}}}=\left(\begin{array}{ll}0 & {\theta }_{{\rm{i}}}\le {\theta }_{{\rm{wp}}}\\ \frac{{\theta }_{{\rm{i}}}-{\theta }_{{\rm{wp}}}}{{\theta }_{{\rm{fc}}}-{\theta }_{{\rm{wp}}}} & {\theta }_{{\rm{wp}}}\le {\theta }_{{\rm{i}}}\le {\theta }_{{\rm{fc}}}\\ 1 & {\theta }_{{\rm{i}}}\ge {\theta }_{{\rm{fc}}}\end{array}\right.$$

14 $${f}_{{\rm{t}}}={e}^{-{\left[\left({T}_{{\rm{a}}}-{T}_{{\rm{opt}}}\right)/{T}_{{\rm{opt}}}\right]}^{2}}$$

15 $${f}_{{\rm{v}}}=\sqrt{VOD/VO{D}_{max}}$$

where *χ* is fractional interception occurring during daytime, which is set to 0.7; *S*_c_ is the water storage capacity of canopy, which is defined as the product of precipitation and *LAI*; *θ*_fc_ is the soil field capacity, and *θ*_wp_ is the soil moisture at the wilting point; *θ*_i_ is the actual soil moisture at the *i*^th^ soil layer; *T*_a_ is the air temperature (*S*_c_); *T*_opt_ is the optimum plant growth temperature (*S*_c_), which can be derived from the air temperature when the multiply of *LAI*, *R*_n_ and *T*_a_ are high; *VOD*_max_ is the maximum value for the annual time series of *VOD* retrievals.

In SiTHv2, the critical threshold of soil moisture (*θ*_c_), which describes the point of the plant under water stress, is dynamically dependent on the plant traits and environmental conditions, rather than a static constant. Hence, the *θ*_c_ can be derived as follows^65,66^:

16 $${\theta }_{{\rm{c}}}=\left(1-p\right)\left({\theta }_{{\rm{fc}}}-{\theta }_{{\rm{wp}}}^{{\rm{h}}}\right)+{\theta }_{{\rm{wp}}}^{{\rm{h}}}$$

17 $$p=\frac{1}{1+{E}_{{\rm{p}}}}-\frac{w}{1+{H}_{{\rm{c}}}}$$

18 $${\theta }_{{\rm{wp}}}^{{\rm{h}}}={\theta }_{{\rm{wp}}}/k$$

where *θ*_fc_ is the soil field capacity; ${\theta }_{{\rm{wp}}}^{{\rm{h}}}$ is the adjusted wilting point soil moisture by considering the canopy height (*H*_c_); *p* is a parameter for regulating *θ*_c_ between *θ*_fc_ and ${\theta }_{{\rm{wp}}}^{{\rm{h}}}$, which can be determined by potential ET (*E*_p_) and *H*_c_; *w* is set to 0.1 denotes the weight of *H*_c_ on the *θ*_c_; *k* is a sensitivity index of soil water content, which can be determined as the square root of *H*_c_. Thus, the soil moisture constraint function for potential transpiration at the *i*^th^ layer (*f*_smv, i_) can be calculated as follows:

19 $${f}_{{\rm{smv}},{\rm{i}}}=\{\begin{array}{ll}0 & {\theta }_{{\rm{i}}}\le {\theta }_{{\rm{wp}}}^{{\rm{h}}}\\ 1-{\left(\frac{{\theta }_{{\rm{c}}}-{\theta }_{{\rm{i}}}}{{\theta }_{{\rm{c}}}-{\theta }_{{\rm{wp}}}^{{\rm{h}}}}\right)}^{k} & {\theta }_{{\rm{wp}}}^{{\rm{h}}}\le {\theta }_{{\rm{i}}}\le {\theta }_{{\rm{c}}}\\ 1 & {\theta }_{{\rm{i}}}\ge {\theta }_{{\rm{c}}}\end{array}$$

Collectively, in the SiTHv2 model, all the water and energy components are theoretically balanced. That is, all the consumption terms (e.g., ET, deep percolation, etc.) and input terms (e.g., precipitation, etc.) are dynamically balanced on the time series. Notably, the dynamic changes in soil moisture at different layers are both intermediate results of the previous time step and important variables regulating the dynamic changes in soil water stress in the next stage. For more detailed model structure, please refer to the authors’ previous studies^20,21^.

### Global data generation

The SiTHv2 model was employed to estimate daily terrestrial ET and its various components at a spatial resolution of 0.1°, as well as the soil moisture dynamics across three soil layers. This model was implemented for each grid cell in their respective time series, driven by forcing data such as air temperature, pressure, net radiation, precipitation, and satellite-based observations of vegetation dynamics and surface albedo. To enhance computational efficiency, parallel computation was implemented at each grid cell. Importantly, a 100-year spin-up period was applied to achieve equilibrium states for each intermediate variable during the simulation. Finally, global seamless estimates of total terrestrial ET, plant transpiration, soil evaporation, canopy intercepted evaporation, and three layers soil moisture were obtained by running the SiTHv2 model from 1982 to 2020. In addition, we also aggregated the daily estimates to provide the monthly and yearly dataset.

## Data Records

The dataset comprises multi-type of outputs, including the total terrestrial ET, plant transpiration (*T*_r_), bare soil evaporation (*E*_s_), canopy interception evaporation (*E*_i_), and the soil moisture dynamics across three soil layers at varying depths. Generated dataset is available at three temporal intervals (i.e., daily, monthly, and annual) and has a global spatial resolution of 0.1°. All data is stored in Network Common Data Form (NetCDF) files, accessible via the data repository of National Tibetan Plateau Data Center (TPDC)^67^. Files with different temporal resolutions are organized in separate directories at TPDC. Filenames follow the structure “SiTH.v2.<VV>.<XXXX>.<YYYY>.nc”, where “VV” represents the variable name (e.g., ET – Evapotranspiration), “XXXX” indicates the temporal resolution (e.g., Daily, Monthly, Annual), and “YYYY” denotes the four-digit year. Further details are provided in the global attributes of each NetCDF file.

## Technical Validation

### ET validation using *in-situ* observations

**Fig. 2:**
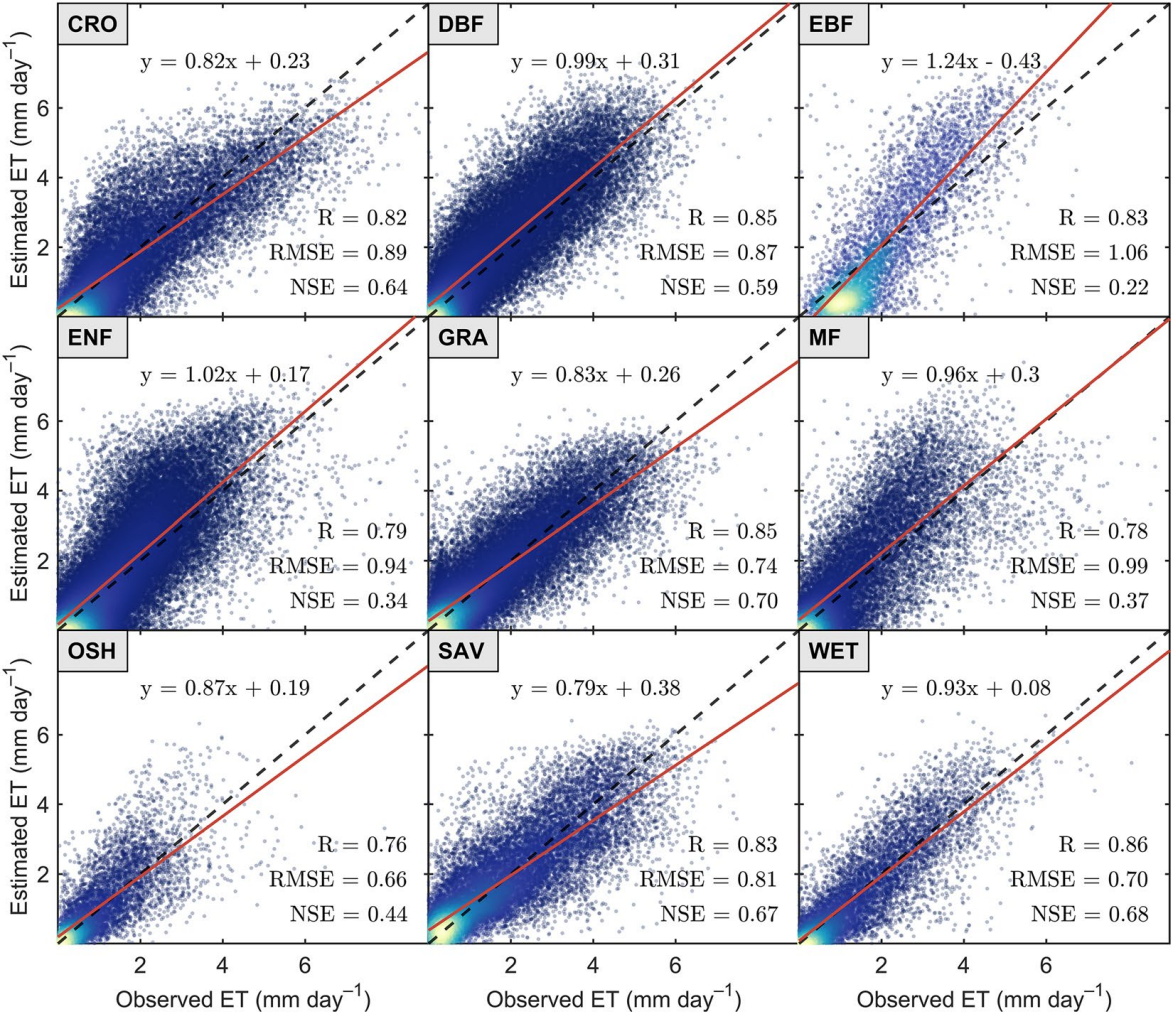
Validation of evapotranspiration (ET) between SiTHv2-derived estimates and *in-situ* observations based on eddy covariance for different plant functional types (PFTs). Details of selected sites are summarized in an Excel file stored in the repository, and the link is available in the Code Availability section.

At the site scale, we employed observed latent heat flux (LE) from the FLUXNET2015 dataset to validate the SiTHv2-derived ET estimates. *In-situ* observed LE flux was converted into ET values by dividing by the latent heat of vaporization. All site observations were categorized according to their plant functional types (PFTs). Figure 2 demonstrates that SiTHv2-derived ET estimates exhibit strong agreement with flux observations, achieving correlation coefficients (*R*) of at least 0.76 across different PFTs. The highest *R*-value, approximately 0.85, is observed in grasslands (GRA), wetlands (WET), and deciduous broadleaf forests (DBF). In terms of root mean square error (RMSE), the largest value occurred in evergreen broadleaf forests (EBF) at 1.06 mm day^−1^, representing the sole situation where RMSE exceeded 1 mm day^−1^ among all PFTs. It is worth noting that RMSE differences are also associated with varying ET magnitudes in different PFTs. Conversely, the dimensionless Nash-Sutcliffe Efficiency (NSE) effectively eliminates this effect. We found that the NSE of SiTHv2-derived ET estimates of EBF is the lowest at 0.22, while it remained greater than 0.5 in other PFTs, even reaching 0.7 in GRA. Additionally, linear slopes between SiTHv2 and *in-situ* observations are closely aligned with the 1:1 line in most PFTs, particularly in DBF (0.99), EBF (1.02), MF (0.96), and WET (0.93). Overall, the SiTHv2 model is capable of providing reliable ET estimates at the *in-situ* level.

### Comparison of ET with independent water balanced ET at basin level

**Fig. 3:**
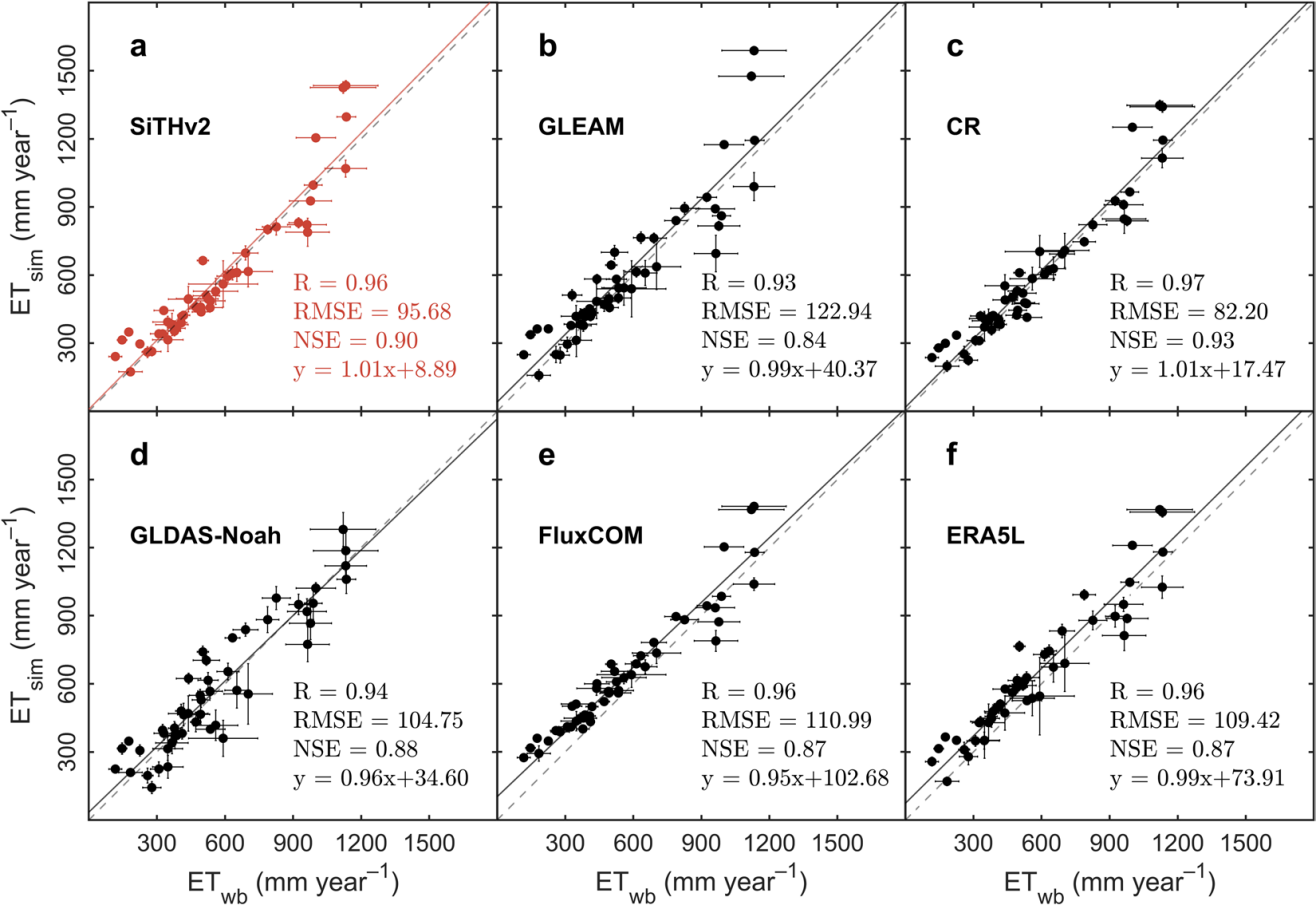
Basin-level comparison between different ET estimates and the independent ET derived from basin water balance across 49 basins. Different sub-panel respectively represented the comparison results from (**a**) the SiTHv2 model; (**b**) the GLEAM model; (**c**) the CR model; (**d**) the GLDAS-Noah; (**e**) the FluxCOM product; (**f**) the ERA5L. The dash line in each sub-figure denotes the 1:1 line. The error bar for each point indicates the annual variation for simulated ET and ETwb, respectively.

We compared the ET estimates derived from SiTHv2 with independent water balance results (*ET*_wb_) across 49 major global basins. This comparison aimed to address uncertainties arising from scale mismatches between *in-situ* measurements and grid-based ET estimations at a spatial resolution of 0.1°. As illustrated in Fig. 3, SiTHv2-derived ET estimates demonstrated a strong agreement with *ET*_wb_, exhibiting a *R*-value of 0.96 and a NSE value of 0.90. Although this NSE value is slightly lower than that of the CR model (NSE = 0.93), both SiTHv2 and CR exhibits identical linear regression slopes of 1.01. Notably, SiTHv2 displayed a superior intercept compared to CR, indicating a closer estimates to *ET*_wb_ in the basins with low ET volumes. While the GLEAM and ERA5L product also showed a close slope (0.99), both of them consistently overestimated ET in comparison to *ET*_wb_ due to their elevated intercepts. In contrast, FluxCOM exhibited the same *R*-value as SiTHv2, however, SiTHv2’s RMSE of 95.68 and NSE of 0.90 surpassed the corresponding FluxCOM statistics, highlighting its better performance at these basins.

**Fig. 4:**
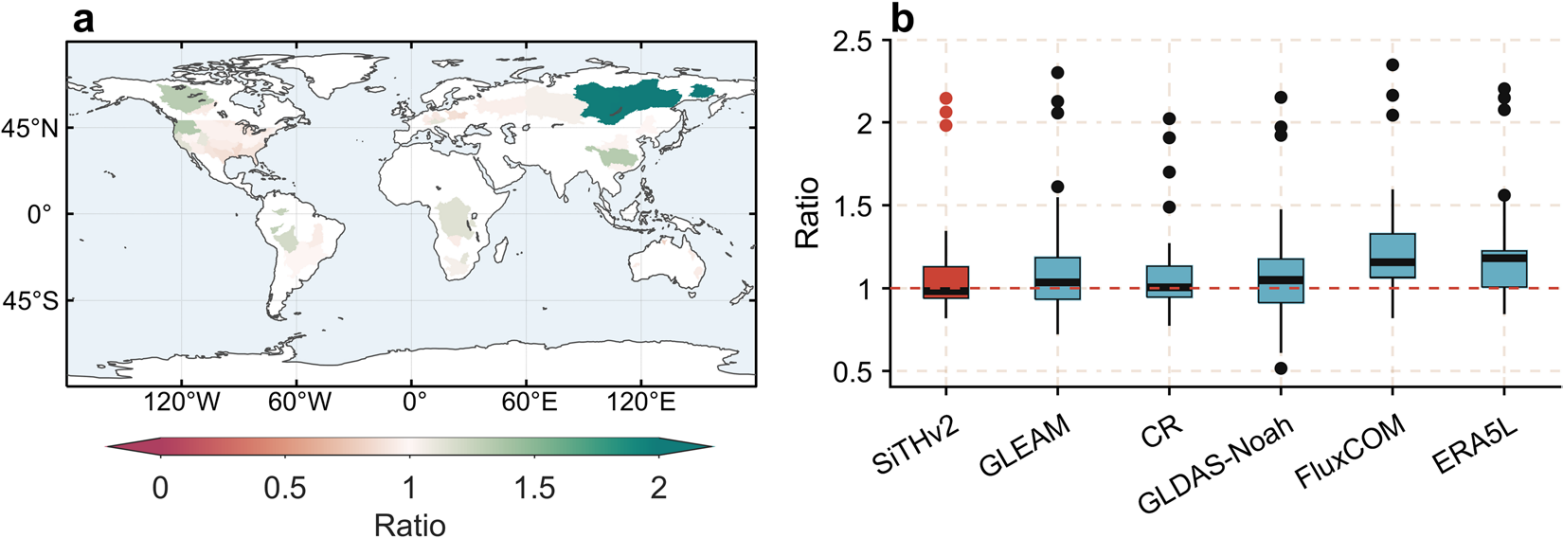
The ratio of multi-year mean ET estimates from different ET datasets relative to the *ET*_wb_ across the 49 basins. (**a**) represents the spatial distribution of the ratio for the SiTHv2; (**b**) is the intercomparison of the ratios for different models/products.

The accuracy of estimated ET volume was further assessed by comparing the ratio of mean annual ET to the *ET*_wb_ across different basins. From Fig. 4a, the majority of basins exhibit ratio values near 1, indicating that the SiTHv2-derived ET is closely aligned with *ET*_wb_. Moreover, a comparison with other mainstream global ET products reveals that the ratio values of SiTHv2 across 49 basins (with a mean value of 0.98) demonstrate superior performance (Fig. 4b). A relatively high ratio value is observed in a few individual basins located at the high latitudes of the Northern Hemisphere, where a similar pattern also found in other ET products. Such discrepancy might be attributed to the inadequate characterization of the soil freezing and thawing process^68,69^ in current ET models.

### Comparison of ET with global ET product

**Fig. 5:**
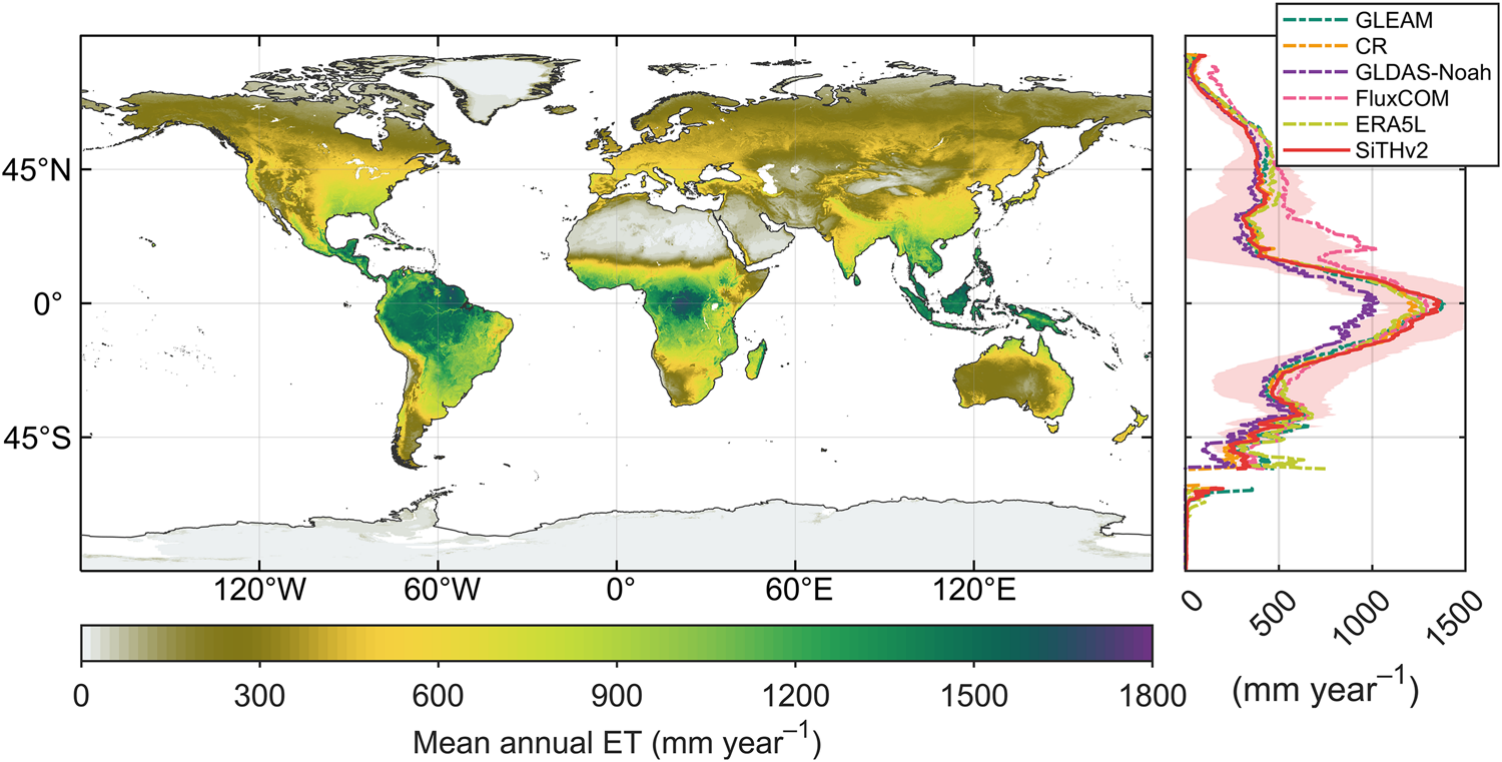
Global distribution of mean annual terrestrial ET derived by the SiTHv2 model, and the comparison of zonally mean profile with the other ET products. The red shadow in the right panel represents the standard deviation for the zonally mean of SiTHv2.

**Fig. 6:**
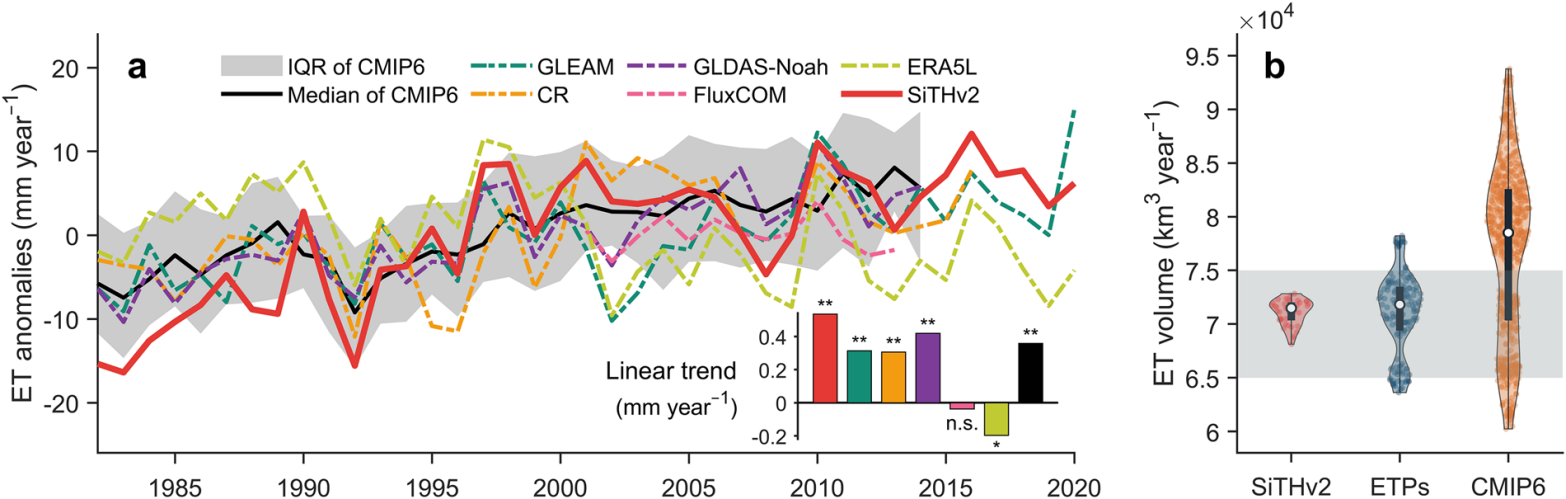
Comparison of annual terrestrial ET from different data sets. (**a**) represents the annual variation and linear trend from 1982 to 2020, where the asterisks denote that the trend passed the Mann-Kendall test with a p-value < 0.01; (**b**) shows the annual total terrestrial ET volume from SiTHv2, mainstream global ET products (ETPs), and the CMIP6 simulations. A total of 20 ensemble ESMs from CMIP6 were used in this study; details can be found in Table S1.

Using the long-term global ET estimates from SiTHv2, we generated a global distribution of mean annual ET (Fig. 5). Spatially, tropical regions exhibit the highest ET, while drylands show the lowest ET. Such pattern is typically determined by the combined effect of atmospheric evaporative demand and local water availability. The zonally mean profile of SiTHv2-derived ET is also similar to other global ET products, particularly the GLEAM and CR. We further evaluated the annual variation of global terrestrial ET. SiTHv2-derived ET displays a variation comparable to most ET products and fall within the interquartile range (IQR) of CMIP6 results (Fig. 6a). Regarding the annual linear trend, most global ET products (except for ERA5L) show a significant increase over the past few decades. SiTHv2 has the highest increasing trend, with a value of 0.53 mm year^−1^ from 1982 to 2020, followed by GLDAS-Noah (0.42 mm year^−1^), and median of 20 ESMs in CMIP6 (0.36 mm year^−1^). CR and GLEAM has the same increasing rate of 0.31 mm year^−1^. It is worth noting that the ET estimates from FluxCOM exhibit an insignificant trend, which due to its shorter time span with covering only the most recent 14 years from 2001. In terms of total volume of global terrestrial ET (Fig. 6b), the mean value of SiTHv2-derived ET is 71.5 × 10^3^ km^3^, closely aligned with the mean value of other ET products (71.8 × 10^3^ km^3^). Comparatively, the 20 ensemble ESMs of CMIP6 present a broader range of global terrestrial ET volume, with a mean value of 78.5 × 10^3^ km^3^. Previous studies^1,50,70–73^ have reported global terrestrial ET volumes primarily within the range of 65 to 75 × 10^3^ km^3^. In summary, the global spatial pattern, annual variation, and total magnitude of ET estimated by SiTHv2 are reliable based on current understandings of global water cycles.

### Comparison of ET partitioning ratio with independent T/ET dataset

**Fig. 7:**
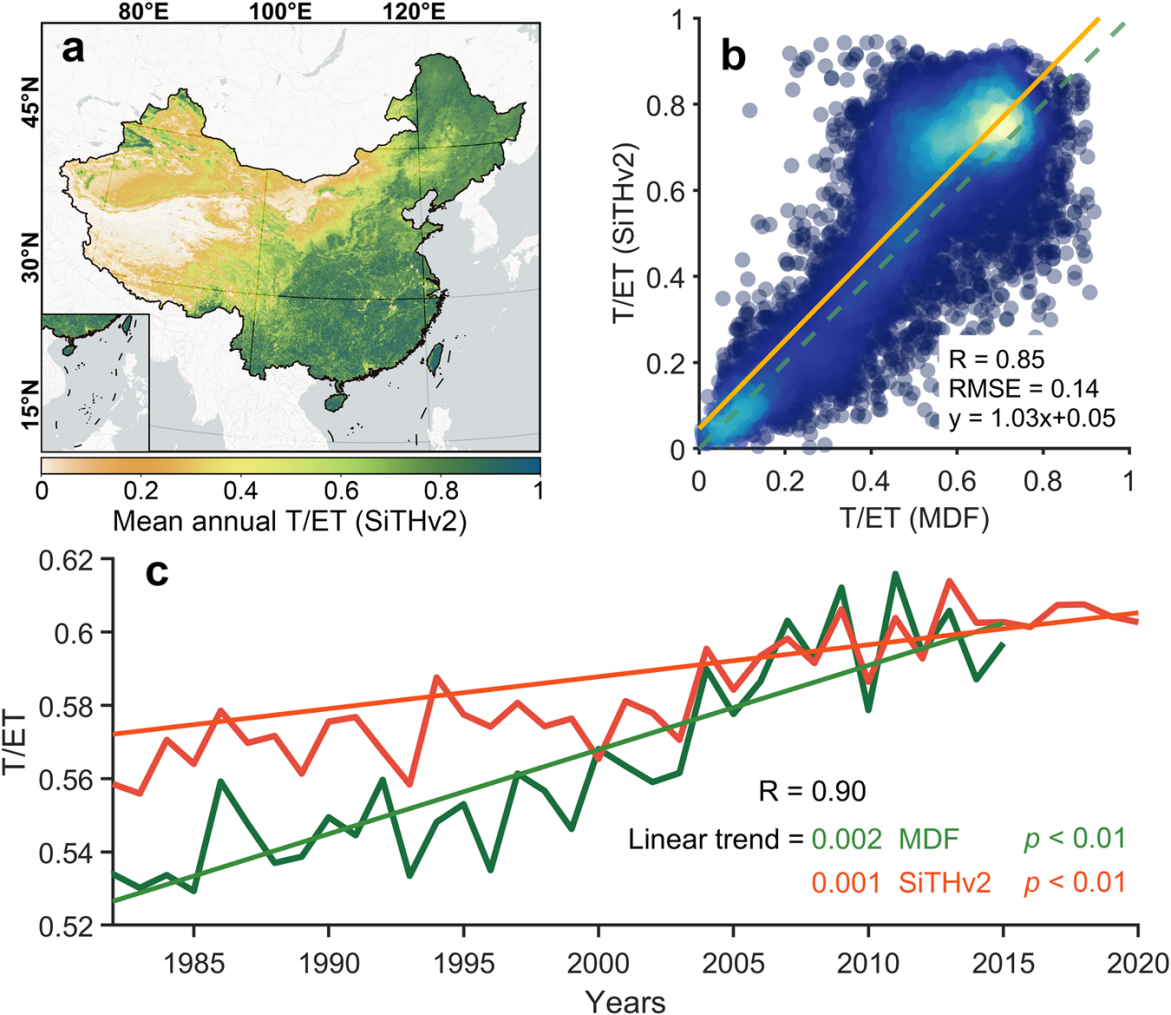
Comparison of spatial patterns and annual variations between SiTHv2- and MDF-derived T/ET ratios over China. (**a**) depicts the spatial distribution of mean annual T/ET across China; (**b**) represents an intensity scatter plot with linear regression based on the grid-wise T/ET from SiTHv2 and MDF; (**c**) displays the annual variation of T/ET in China from SiTHv2 and MDF, respectively. The significance of the linear trend is based on the Mann-Kendall test with a p-value < 0.01.

The SiTHv2 model enables estimation of different components of total ET, making the ratio between transpiration and ET (T/ET) a crucial metric for evaluating accuracy of ET partitioning^74^. Although *in-situ* measurements can provide the T/ET benchmark based on sap flow and eddy covariance techniques, their relative scarcity and short duration limit the provision of grid-based reference applied globally^75^. Moreover, isotope-based methods, which also only cover short measurement periods, tend to overestimate T/ET^27,76,77^. By contrast, Model-data fusion (MDF) method can be employed to incorporate available observations into a process-based framework. A recent study published a new T/ET dataset over China spanning from 1981 to 2015, based on multivariate observations, demonstrating good agreement with independent site measurements^78^. Hence, we extracted the grid-based T/ET ratios from SiTHv2 across China and compared with the MDF product (Fig. 7). The results indicate that the SiTHv2-derived T/ET ratios exhibit not only high spatial agreement with MDF, with a *R*-value of 0.85, RMSE is 0.14, and regression slope is 1.03 with intercept of 0.05 (Fig. 7b), but also a strong correlation for long-term variations (*R* = 0.90). Additionally, the linear trend for T/ET estimates from SiTHv2 and MDF are both significant (*p* < 0.01), with a value of 1 × 10^−3^ year^−1^ for SiTHv2 and 2 × 10^−3^ year^−1^ for MDF.

### Soil moisture validation using *in-situ* observations

**Fig. 8:**
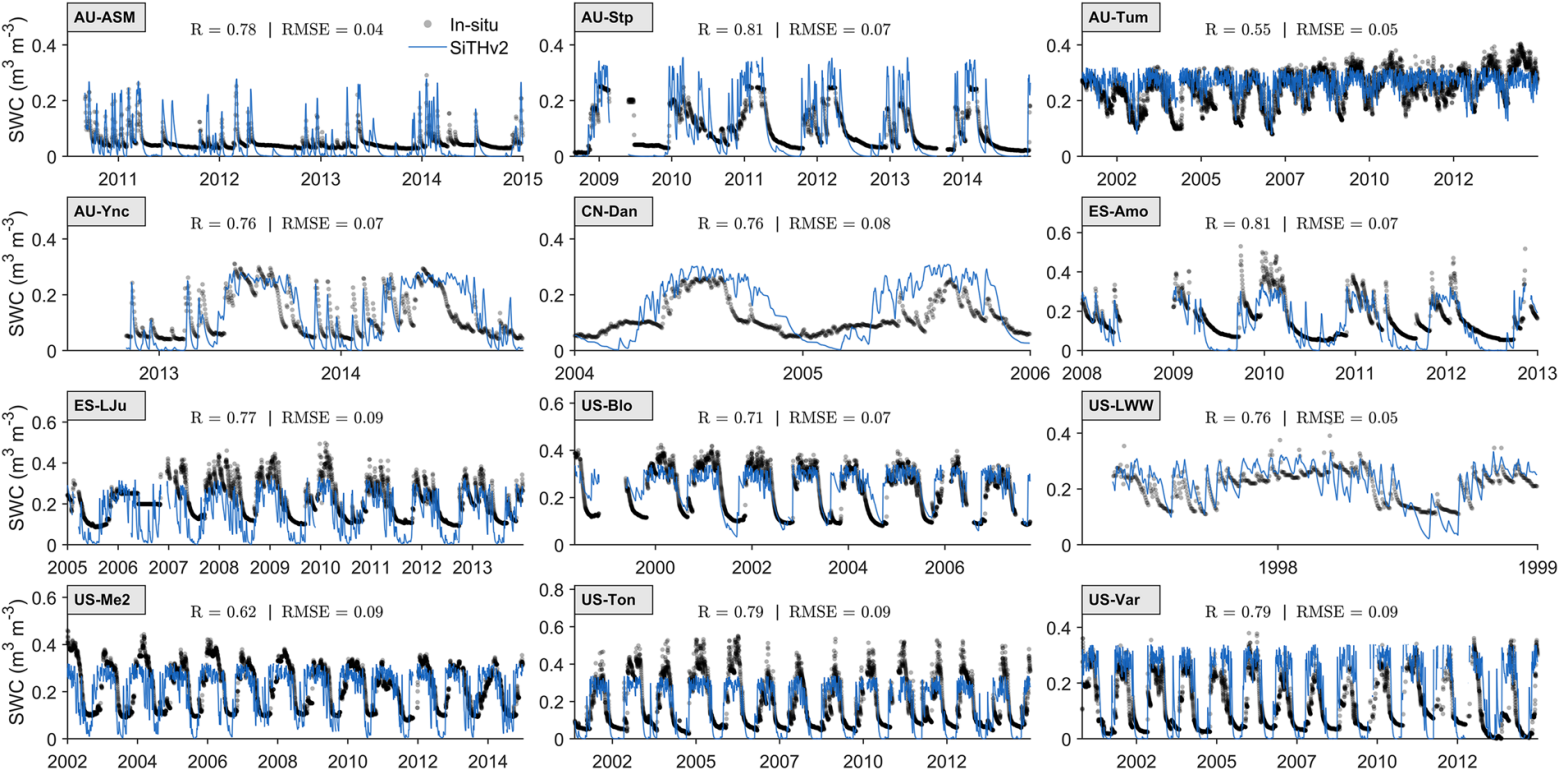
Validation of soil moisture estimates (layer 1) obtained from the SiTHv2 model based on daily-scale surface soil moisture observations at 12 stations. The time span for different sites is adjusted according to the observation duration at each site.

Soil moisture, also known as soil water content (SWC), is a key variable in the SiTHv2 model. Due to the limited availability of SWC measurements at most flux stations, we selected 12 sites with observed SWC to validate the performance of the SWC estimates in SiTHv2 model at the *in-situ* level. These selected flux sites can cover the majority of various PFTs under different climate conditions. As depicted in Fig. 8, the SiTHv2 model can generally capture the dynamic changes in SWC across most sites. Specifically, the correlation coefficient reaches a mean value of 0.75, with the highest *R*-value of 0.81 observed at the AU-Stp (GRA) while the lowest *R*-value of 0.55 at the AU-Tum (EBF). The metrics of RMSE for all selected sites are relatively small, with a mean value of 0.07 m^3^ m^−3^. The site AU-ASM (SAV) has the smallest RMSE value of 0.04 m^3^ m^−3^, while four sites (i.e., ES-LJu, US-Me2, US-Ton, and US-Var) exhibit a slightly higher RMSE value of 0.09 m^3^ m^−3^. In addition to the internal error of model performance, it should be noted that the differences in SWC depth between the SiTHv2 model (first layer at 5 cm) and site measurements (depth varying across sites) may also contribute to uncertainties in such comparisons. Nonetheless, the *in-situ* comparisons indicate that the SWC estimates from the SiTHv2 model are reliable and successfully characterize soil moisture dynamics across different sites globally.

### Comparison of soil moisture with data-driven product

**Fig. 9:**
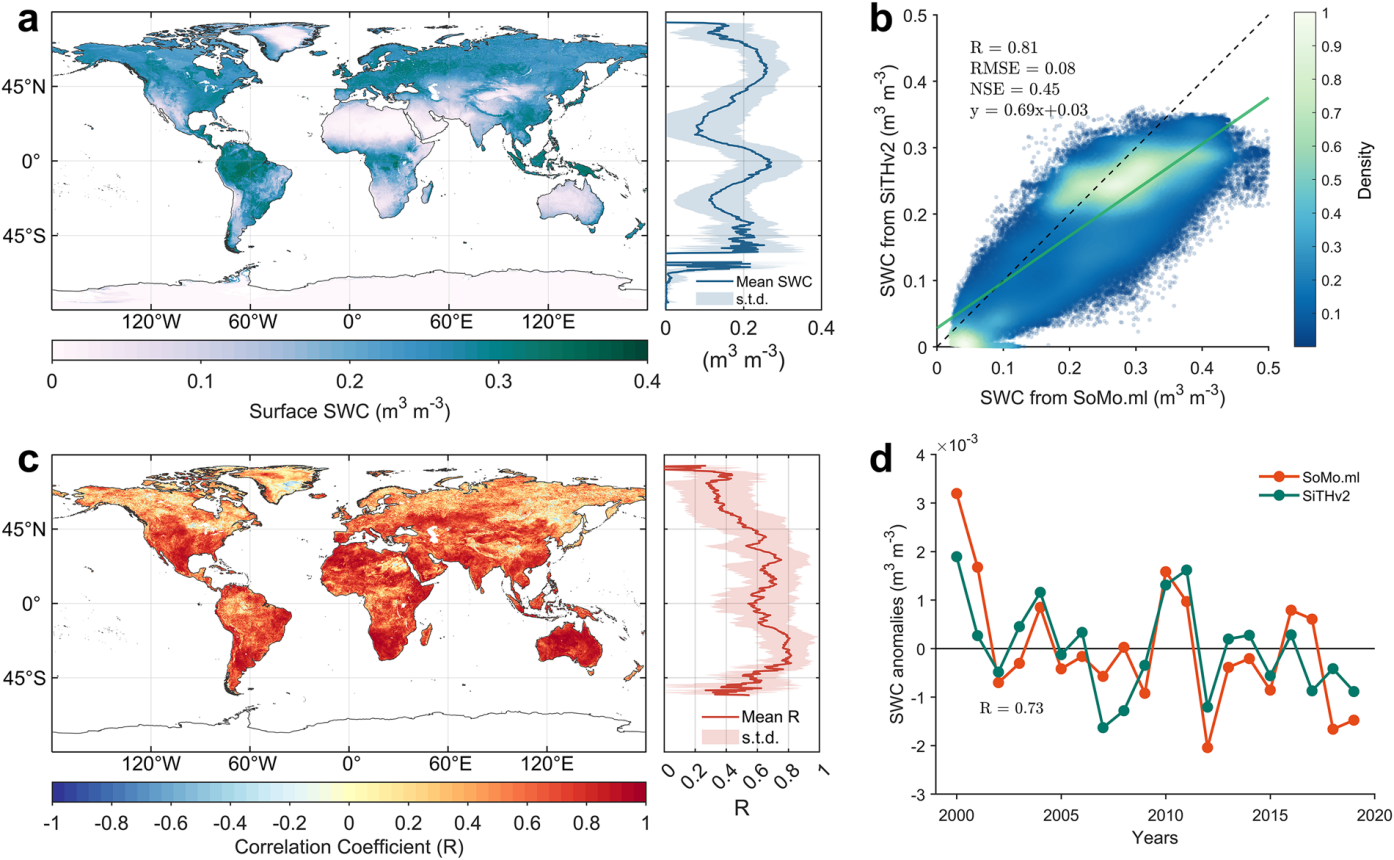
Soil moisture estimate comparison between SiTHv2 and SoMo.ml. (**a**) Mean annual surface soil water content (SWC) from SiTHv2 spanning 1982 to 2020; (**b**) Mean annual spatial pattern regression between SiTHv2 and SoMo.ml during the overlapping period of 2000 to 2019; (**c**) Global correlation between SiTHv2 and SoMo.ml at each grid cell; (**d**) Annual variation of global SWC.

To evaluate the global spatial pattern and magnitude of SiTHv2-derived SWC estimates, we employed the recently published global soil moisture product, SoMo.ml^58^, which is built upon deep learning techniques (i.e., LSTM network) and numerous *in-situ* observations. Figure 9a illustrates the mean annual surface SWC spanning from 1982 to 2020, with tropical areas exhibiting the highest SWC and the high latitudes of Northern Hemisphere following closely. Excluding polar regions, zones surrounding 20°N and 20°S displays the lowest zonally mean SWC, attributed to the vast drylands and deserts in these areas. Furthermore, the surface SWC estimated by SiTHv2 shows good spatial agreement with SoMo.ml (Fig. 9b), with a *R*-value of 0.81 and an overall RMSE of 0.08 m^3^ m^−3^. A high NSE value (0.45) also suggests high reliability when compared to SoMo.ml. Meanwhile, we analyzed the correlation between SiTHv2- and SoMo.ml-derived annual SWC at each grid cell (Fig. 9c), revealing that a large portion of the globe presents a high *R*-value, particularly exceeding 0.60 for the areas between 45°N and 45°S. Additionally, the annual variations of SWC in these two datasets are quite similar, achieving a *R*-value of 0.73. It is important to note that the comparison was conducted from 2000 to 2019, constrained by the temporal range of SoMo.ml. Overall, SiTHv2-based SWC estimates not only exhibit reasonable spatial patterns but also supply reliable annual dynamics across the majority of grid cells on a global scale.

### Comparison of soil moisture with satellite product

**Fig. 10:**
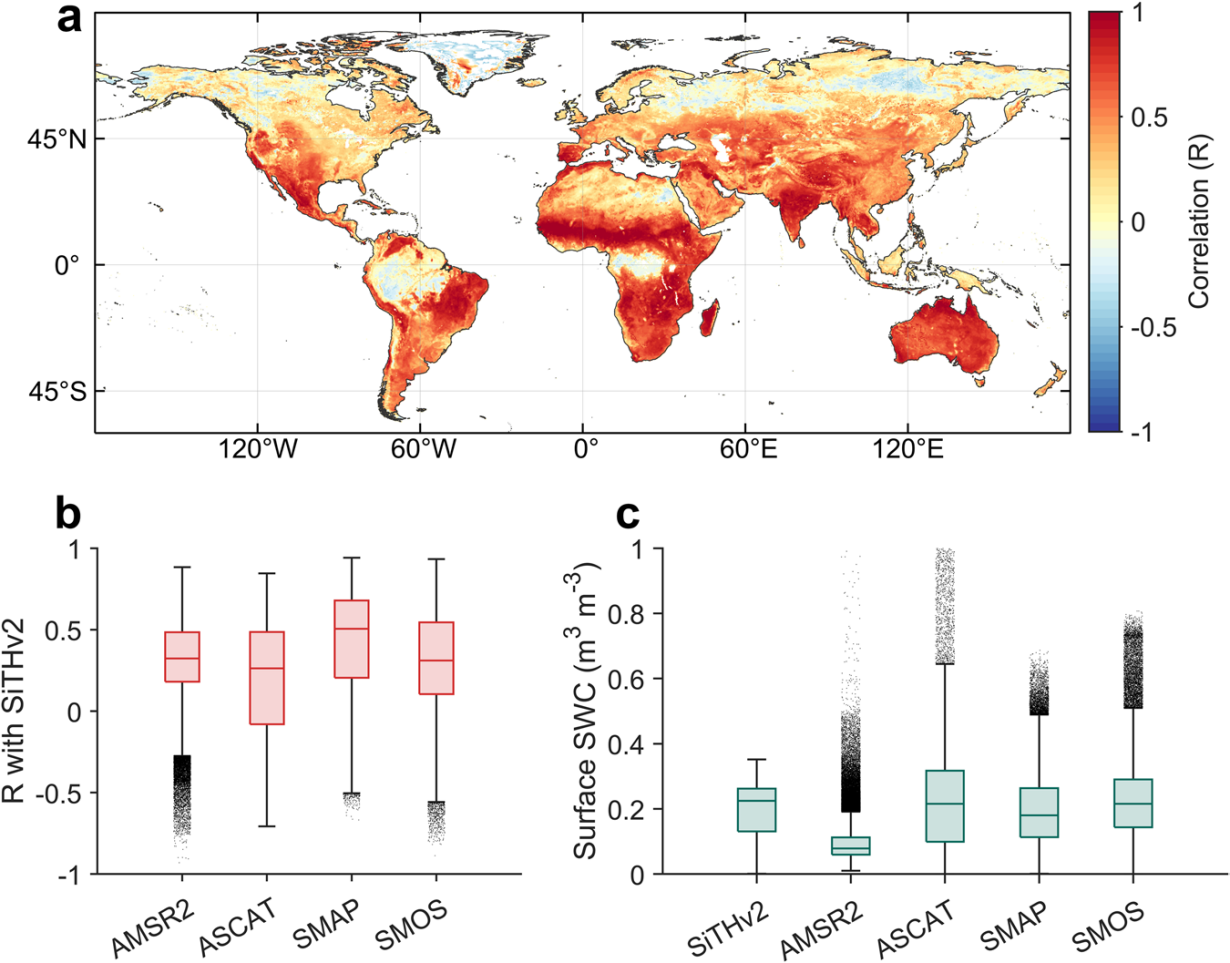
Overall comparison of SWC derived from SiTHv2 with satellite-based products. (**a**) Spatial distribution of the mean correlation coefficient (R) between annual SWC from SiTHv2 and four satellite-based products (AMSR2, ASCAT, SMAP, and SMOS); (**b**) Boxplot shows the global statistics of R values for each combination between SiTHv2-SWC and different satellite-based SWC products; (**c**) The range distribution of SWC magnitudes from SiTHv2 and other satellite-based products.

In recent years, microwave remote sensing technology has become crucial for monitoring large-scale surface soil moisture via satellites, resulting in various satellite-based soil moisture products. Despite most products covering only the past dozen years due to the satellite launch, microwave remote sensing, based on the physical mechanism of dielectric constant, is currently the most direct and effective way for obtaining large-scale surface soil water conditions. It also serves as an important benchmark for evaluating the SWC estimates at a large scale. We compared surface SWC from SiTHv2 with four microwave-based SWC products: SMAP, SMOS, ASCAT, and AMSR2. SiTHv2-SWC shows high consistency with satellite-SWC products globally, particularly in arid and semi-arid regions, where the mean correlation coefficient (*R*) can reach 0.8 or higher (Fig. 10a). Notably, lower *R*-values are observed in tropical rainforests (e.g., Amazon) and high-latitude regions (above 60°N). This does not imply significant errors in SiTHv2-derived SWC in these areas, as microwave retrievals of surface soil moisture are affected by factors such as dense vegetation and soil freeze-thaw processes, causing signal interference and larger errors in satellite-SWC accuracy in regions with forest and snow cover^79^. Among the four satellite-based SWC products, SiTHv2 exhibits the highest correlation with SMAP, with a median *R*-value over 0.5 based on grid-by-grid statistics (Fig. 10b). Furthermore, the range of SWC values for SiTHv2 in terms of the 25th to 75th percentile is closer to SMAP and SMOS products, while AMSR2 produces the lowest results (Fig. 10c).

**Fig. 11:**
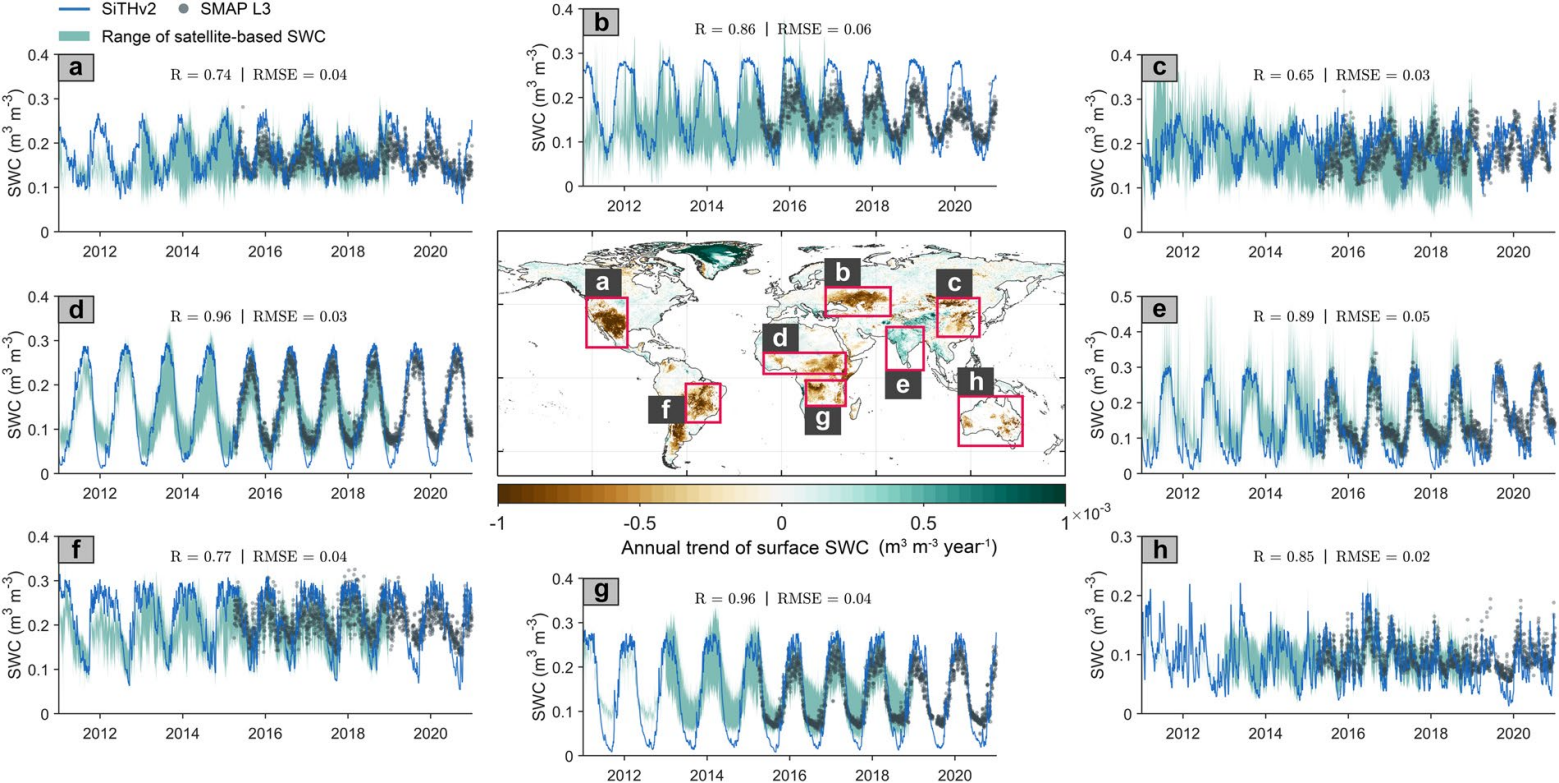
Global pattern of the annual trend in SWC derived from SiTHv2 from 1982 to 2020; The subplots depict temporal comparisons (daily) between SiTHv2- and satellite-based SWC products in selected hotspot regions, include: (**a**) western United States; (**b**) Central Asia; (**c**) North China; (**d**) southern Sahara; (**e**) Indian subcontinent; (**f**) Brazilian Plateau; (**g**) southern Africa; and (**h**) Australia. The temporal variation of satellite-based SWC products starts from 2011, subject to the data availability (e.g., SMOS). The SMAP L3 data is highlighted with black dots, while the remaining satellite products are presented as a range of ensemble mean.

In addition, we calculated the interannual trend of surface SWC from 1982 to 2020 using the SWC estimates from SiTHv2. The global spatial distribution of the annual trend is displayed in Fig. 11, revealing considerable spatial differences in SWC changes. For example, soil moisture has notably increased in the Indian subcontinent and the Tibetan Plateau, while arid and semi-arid regions like the western United States and southern Sahara have become increasingly dry. We selected eight typical regions with significant SWC increases or decreases over the past 39 years (1982–2020) for a daily-scale comparison of SiTHv2-derived SWC and other satellite products. Statistically, SiTHv2 effectively captures SWC seasonality in these key regions, with *R*-value of at least 0.65 (Fig. 11c) and up to 0.96 (Fig. 11d,g), while RMSE values range from a maximum of 0.06 m^3^ m^−3^ (Fig. 11b) to a minimum of 0.02 m^3^ m^−3^ (Fig. 11h). Overall, the SiTHv2-derived SWC estimates closely align with microwave-based satellite observations, particularly showing higher consistency with the SMAP L3 product. Thus, the comparison with satellite products, combined with previous SoMo.ml statistics, collectively suggest that the global SWC estimates from SiTHv2 are reliable and can provide robust data support for hydrology- and climate-related applications.

## Usage Notes

The original datatype running in the SiTHv2 model is the 32-bit double float, while the outputs are stored as 16-bit short integers with two decimal places to conserve storage space and facilitate transfer and usage. Consequently, the released dataset must be rescaled to obtain corrected values for each variable. Specifically, high-level software (e.g., Matlab, ArcGIS, etc.) or programming languages (e.g., Python, R, NCL, etc.) can be utilized to load data from the NetCDF file. Subsequently, the “scale_factor” attribute for each variable should be applied to retrieve the corrected values as follows:

20 $$Va{r}_{{\rm{corrected}}}={{\rm{double}}}_{-}{\rm{float}}(Va{r}_{{\rm{original}}})\times {{\rm{scale}}}_{-}{{\rm{factor}}}^{-1}$$

Although the newly SiTHv2-derived ET and soil moisture product perform well compared to similar data products, there are still limitations and uncertainties remain due to errors in forcing data and internal simulation processes. For instance, precipitation serves as a crucial water input in the SiTHv2 model, significantly influencing soil moisture and water supply for ET. However, current precipitation datasets, including the ERA5L precipitation used in this study, exhibit high uncertainties stemming from a scarcity of ground-based observations in certain regions^80^. Additionally, the model does not emphasize the freeze-thaw process of soil at low temperatures in high latitudes, which may introduce errors in soil moisture (ice and water) during boreal winters^68,69^, although the absolute ET amount does not undergo major changes at such low temperatures. Nonetheless, based on multi-scale validation and comparison, the SiTHv2-derived product is capable of offering reliable ET and soil moisture estimates. By featuring long-term time span and global seamless coverage with high spatio-temporal resolution, this dataset will provide a dependable solution and data support for global water cycle analysis, drought assessment, energy budget, and climate change related studies.

### Supplementary information

Supplementary Information

## Data Availability

The codes to process the data and generate the figures, and the details of selected flux sites are available at https://github.com/kunlz/Codes.longterm.SiTHv2.product. The model codes of SiTHv2 are available at https://github.com/kunlz/SiTHv2.
