# Supplementary material for: A global dataset of terrestrial evapotranspiration and soil moisture dynamics from 1982 to 2020
Source: Sci Data. 2024 May 3;11:445. doi: 10.1038/s41597-024-03271-7 (PMC11068785; doi:10.1038/s41597-024-03271-7)
Supplement: Supplementary file 1 — Supplementary Information [file 41597_2024_3271_MOESM1_ESM.docx]

Supplementary Information

**A global dataset of terrestrial evapotranspiration and soil moisture dynamics from 1982 to 2020**

Kun Zhang, Huiling Chen, Ning Ma, Shasha Shang, Yunquan Wang, Qinglin Xu, Gaofeng Zhu

**Contents of this file**

1. Figure S1

2. Table S1

**
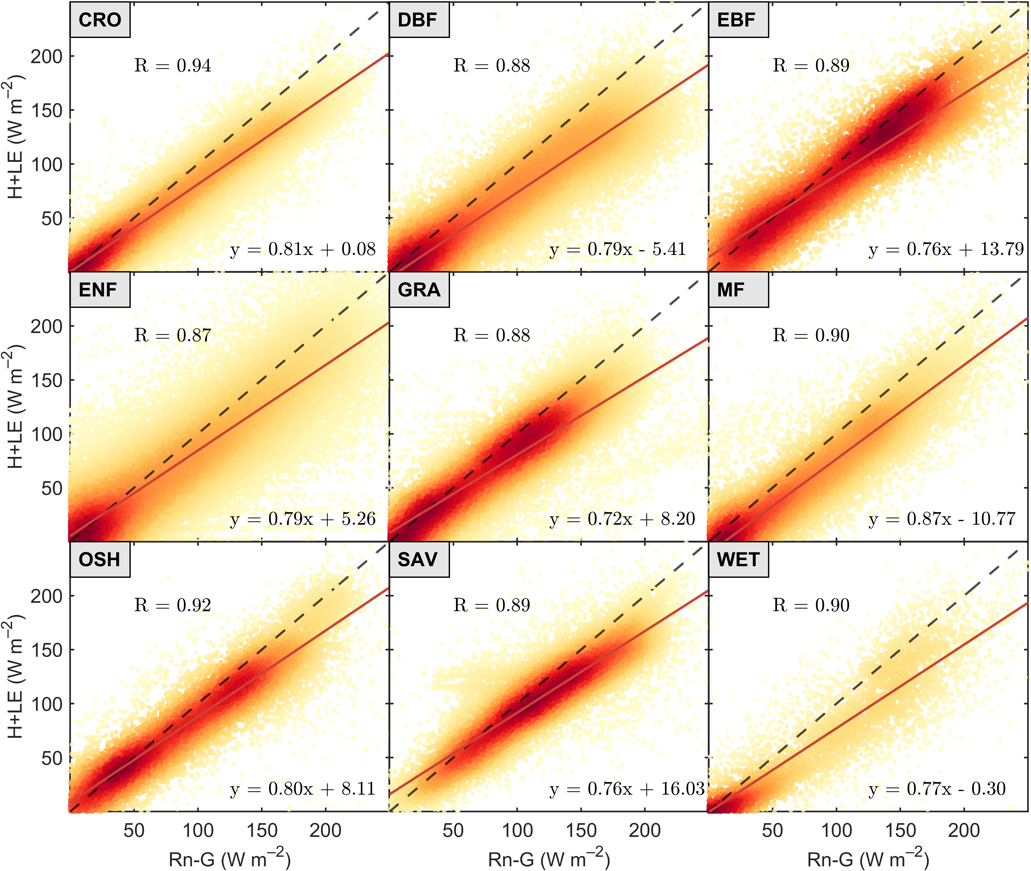
**

**Figure S1.** The energy closure evaluation is conducted for flux data of each plant functional type (PFT) used in this study.

**Table S1.** List of selected 20 Earth System Models in the CMIP6

| Model | Description |
| --- | --- |
| ACCESS-CM2 | Australian Community Climate and Earth System Simulator coupled model |
| AWI-CM | Alfred Wegener Institute Climate Mode |
| BCC-CSM | Beijing Climate Center Climate System Model |
| CAMS-CSM | Chinese Academy of Meteorological Sciences Climate System Model |
| CanESM5 | The Canadian Earth System Model version 5 |
| CAS-ESM2.0 | The second version of the Chinese Academy of Sciences Earth System Model |
| CESM2 | Community Earth System Model Version 2 |
| CESM2-WACCM | The CESM2 uses atmosphere component from the Whole Atmosphere Community Climate Model |
| CMCC-CM2 | Centro Euro-Mediterraneo per Cambiamenti Climatici Climate Model 2 |
| FGOALS-g3 | The Flexible Global Ocean-Atmosphere-Land System Model Grid-Point Version 3 |
| FIO-ESM | The First Institute of Oceanography Earth System Model |
| GISS-E2-1 | The Goddard Institute for Space Studies Model-E 2.1 |
| IITM ESM | The Indian Institute of Technology Madras Earth System Model |
| MCM-UA-1-0 | University of Arizona - Department of Geosciences (UA) MCM-UA-1-0 model |
| MIROC6 | The sixth version of the Model for Interdisciplinary Research on Climate |
| MPI-ESM1-2 | The Max Planck Institute for Meteorology Earth System Model version 1.2 |
| MRI-ESM2-0 | The Meteorological Research Institute Earth System Model Version 2.0 |
| NESM3 | The NUIST Earth System Model version 3 |
| NorESM2 | The second version of the fully coupled Norwegian Earth System Model |
| SAM0-UNICON | Seoul National University atmosphere model version 0 with a unified convection scheme |
